# Optofluidic Formaldehyde Sensing: Towards On-Chip Integration

**DOI:** 10.3390/mi11070673

**Published:** 2020-07-10

**Authors:** Daniel Mariuta, Arumugam Govindaraji, Stéphane Colin, Christine Barrot, Stéphane Le Calvé, Jan G. Korvink, Lucien Baldas, Jürgen J. Brandner

**Affiliations:** 1 Institute of Microstructure Technology, Campus Nord, Hermann-von-Helmholtz-Platz 1, 76344 Eggenstein-Leopoldshafen, Germany; daniel.mariuta@kit.edu (D.M.); arumugam_g@outlook.com (A.G.); jan.korvink@kit.edu (J.G.K.); 2 Institut Clément Ader (ICA), Université de Toulouse, CNRS, INSA, ISAE-SUPAERO, Mines-Albi, UPS, 31400 Toulouse, France; stephane.colin@insa-toulouse.fr (S.C.); christine.barrot@insa-toulouse.fr (C.B.); 3 Université de Strasbourg, CNRS, ICPEES UMR 7515, F-67000 Strasbourg, France; slecalve@unistra.fr; 4 In’Air Solutions, 25 rue Becquerel, 67000 Strasbourg, France

**Keywords:** metal-oxide-semiconductor (CMOS)-based fluorescence sensing, light emitting diode (LED)-induced fluorescence, SU-8 2015 waveguide, silicon fluidic cell, 3,5–diacetyl-1,4-dihydrolutidine (DDL)

## Abstract

Formaldehyde (HCHO), a chemical compound used in the fabrication process of a broad range of household products, is present indoors as an airborne pollutant due to its high volatility caused by its low boiling point (T=−19 °C). Miniaturization of analytical systems towards palm-held devices has the potential to provide more efficient and more sensitive tools for real-time monitoring of this hazardous air pollutant. This work presents the initial steps and results of the prototyping process towards on-chip integration of HCHO sensing, based on the Hantzsch reaction coupled to the fluorescence optical sensing methodology. This challenge was divided into two individually addressed problems: (1) efficient airborne HCHO trapping into a microfluidic context and (2) 3,5–diacetyl-1,4-dihydrolutidine (DDL) molecular sensing in low interrogation volumes. Part (2) was addressed in this paper by proposing, fabricating, and testing a fluorescence detection system based on an ultra-low light Complementary metal-oxide-semiconductor (CMOS) image sensor. Two three-layer fluidic cell configurations (*quartz*–SU-8–quartz and *silicon*–SU-8–quartz) were tested, with both possessing a 3.5 µL interrogation volume. Finally, the CMOS-based fluorescence system proved the capability to detect an initial 10 µg/L formaldehyde concentration fully derivatized into DDL for both the quartz and silicon fluidic cells, but with a higher signal-to-noise ratio (*SNR*) for the silicon fluidic cell ($SNR_{silicon}=6.1$) when compared to the quartz fluidic cell ($SNR_{quartz}=4.9$). The signal intensity enhancement in the silicon fluidic cell was mainly due to the silicon absorption coefficient at the excitation wavelength,$\;a\left(\lambda_{abs}=420\;\mathrm{nm}\right)=5\times 10^{4}{\;\mathrm{cm}}^{-1}$, which is approximately five times higher than the absorption coefficient at the fluorescence emission wavelength, $a\left(\lambda_{em}=515\;\mathrm{nm}\right)=9.25\times 10^{3}{\;\mathrm{cm}}^{-1}$.

## 1. Introduction

Indoor air pollutant concentrations are known to be up to five times higher than outdoors. People generally spend 90% of their time indoors, and approximately 3.8 million people die yearly from diseases related to the exposure to indoor pollution [1]. Among all the indoor pollutants, volatile organic compounds (VOCs) are of particular interest due to their high levels of toxicity [2]. One of the VOCs that raises increased concern is formaldehyde (HCHO), an allergenic, mutagenic, and carcinogenic compound [3,4]. HCHO is largely used in the fabrication process of building materials, household products, and resins for wood products. There are ongoing research projects looking for a non-harmful HCHO alternative in the form of bio-based platform chemical 5-HMF (5-Hydroxymethylfurfural) [5], but meanwhile industrial consumption of HCHO is constantly increasing.

Indoor HCHO concentrations are up to fifteen times higher than those measured outdoors [6] and may have values ranging from 10 to 100 µg/m^3^ (1 to 82 parts-per-billion (ppb) at 25 °C and atmospheric pressure) [7]. The World Health Organization (WHO) standard for a safe daily exposure is set to 100 µg/m^3^ (82 ppb) concentration for a maximum period of 30 min. In France, the recommendations are to limit the exposure to 30 µg/m^3^ (25 ppb), which will be reduced from 2023 onwards to 10 µg/m^3^ (8 ppb) [8,9]. The United States Environmental Protect Agency (US EPA) relies on a 2,4-dinitrophenylhydrazine (DNPH) tube sample followed by offline laboratory high-performance liquid chromatography (HPLC) analysis as the standard HCHO detection methodology [10]. This method exhibits the lowest detection limit of 0.048 µg/m^3^ (0.04 ppb), but it provides average concentrations over time intervals ranging from one hour to a week for both active and passive samplings, respectively [9,10].

Alternative detection methods have been developed in order to enable real-time and in-field HCHO detection, such as proton transfer reaction mass spectrometry (PTR-MS) [11], gas chromatography mass spectrometry (GC-MS) [12], infrared diode laser spectroscopy [13,14], and Hantzsch reaction monitoring [15]. These methods rely on bulky instruments that are not or barely portable, and are, therefore, not compatible with indoor air monitoring. A device adapted for this need should be real-time, selective, palm-held, low-noise, battery-operated, and cost-effective [7,15].

**Figure 1:**
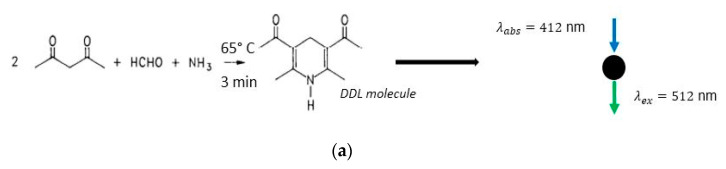

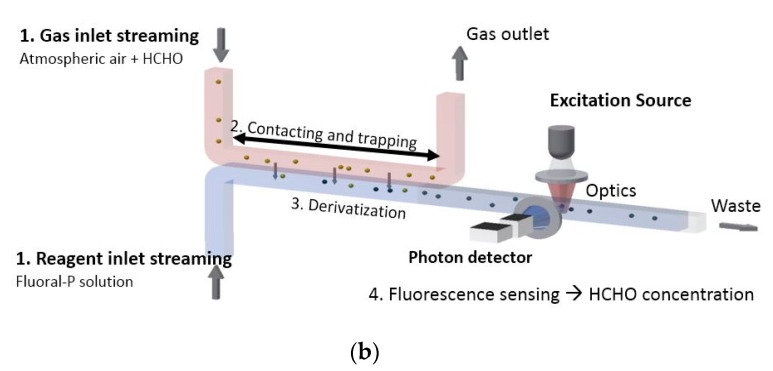
Formaldehyde (HCHO) detection methodology. (**a**) Chemical derivatization reaction of formaldehyde into 3,5–diacetyl-1,4-dihydrolutidine (DDL). (**b**) Gas–liquid contacting, trapping, and detection scheme.

Among the methods used for online HCHO sensing, the method based on the Hantzsch reaction coupled to fluorescence optical detection (see Figure 1a) is known as the most sensitive and selective [10,15]. The continuous detection methodology can be briefly described as a sequence of four steps: (1) air and reagent streaming; (2) HCHO trapping; (3) derivatization into a fluorescent compound; and (4) optofluidic fluorescence detection [9]. During step (1), the air and reagent phases are continuously put in contact, with the fluids being pressure-driven into the system using micropumps. During step (2), the HCHO molecules from the air are trapped in a 4-amino-3-penten-2-one (Fluoral-P) or acetylacetone solution. The molecular transfer from the gaseous phase to the liquid phase is characterized by a convection–diffusion mechanism [16]. The efficiency of the diffusion process is strictly related to a couple of parameters, such as the contacting area between the two phases, the contacting time, and the molecular driving forces across the gas–liquid interface (e.g., partial pressure difference, chemical potential, concentration difference) [17]. During step (3), a full derivatization process of the HCHO molecule into the 3,5–diacetyl-1,4-dihydrolutidine (DDL) fluorescent molecule takes place for a residence time of t=3 min at T=65 °C (see Figure 1b).

Some works related to on-chip optofluidic integration of HCHO detection in either food [18,19,20], Chinese herbs [21], or indoor air [7,22] have recently been reported. A microfluidic paper-based analytical device (µPAD) was proposed by Guzman et al. [18] for detection of low HCHO concentrations in food. The detection was made using a complementary metal-oxide-semiconductor (CMOS) camera and the concentration was found by interpreting the images based on RGB color analysis software on a smartphone. The detection mechanism worked offline and the formaldehyde detection range of the device was 0.2–2.5 mg/L. Improvements in terms of portability, response time, and detection range of 0–0.8 mg/L for this system was presented by Liu et al. [19]. Weng et al. [20] integrated four reaction reservoirs and one substrate reservoir on a polydimethylsiloxane (PDMS) microfluidic chip, enabling HCHO detection based on the absorption spectroscopy in 2 µL food samples. A three-layer poly (methyl methacrylate) (PMMA) device with a detection range of 1–50 mg/L was presented by Liu et al. [21] for the detection of HCHO concentrations in Chinese herbs; it was based on laser-induced fluorescence.

A microfluidic lab-on-a-chip derivatization technique based on the GC-MS measurement technique for HCHO indoor air sensing was described by Pang et al. [22]. The method relied on a glass Pyrex microreactor with a 2.0 m long and 620 µm internal diameter round microchannel as a reactor. The reaction of HCHO with two reagents (pentafluorophenyl hydrazine and O-(2,3,4,5,6-pentafluorobenzyl) hydroxylamine) was studied in a microfluidic context, with the system achieving continuous sampling and analysis with a time resolution of 30 min and a limit of detection (LOD) down to 1.2 µg/m^3^ (1 ppb). The reagent flow rates were varied between 20 and 120 µL/min, while the maximum operating temperature and pressure were  T=300 °C and p=3 MPa, respectively. Despite the evident advantages of low reactant flow rates, the operating parameters and the time resolution were too high to be compatible with the previously mentioned performances of a HCHO palm-held detector.

Becker et al. [7] developed a microfluidic analytical device (20 cm × 25 cm × 15 cm) dedicated to air analysis based on the Hantzsch reaction, which reached a LOD of 0.13 µg/m^3^ (0.1 ppb) for a 17 µL/min reagent flow rate in a microporous tube (10.0 cm length, 0.9 mm internal diameter). The porous tube was placed in a gas microchamber fueled with gaseous HCHO at a flow rate of 250 NmL/min. The temporal resolution was 2 s and the response time was 15 min, enabling near-real-time detection on a portable device. The system performance might be enhanced by simplifying the fluorescence detection system based on a photomultiplier tube (PMT) detector and by reducing the interrogation volume.

Even if the Hantzsch-reaction-based method relies on “wet chemistry”, involving a continuous reagent consumption, this method is currently one of the most reliable candidates for developing a sensitive palm-held continuous detector. The alternatives to the wet-chemistry-based methods are electrochemical detection methods. The recently developed electrochemical sensors, despite their rapid response, operate at very high temperatures, are not energy efficient, and cannot be deployed in atmospheres with flammable potential [23]. Their main disadvantages are their low sensitivity and the absence of selectivity; other aldehydes and gas pollutants can interfere with the formaldehyde measurements, as observed by Baldelli et al. [24].

Thus, the aim of developing a palm-held sensitive continuous detector could be achieved by exploiting the recent advancements in the fields of microfluidics, optofluidics (combining microfluidics and optics), microfabrication, and integrated system technology. On-chip integration of microfluidic analytical applications poses multidisciplinary challenges, generally related to: (1) fluid–fluid interactions; (2) fluid–structure interactions; (3) microstructure fabrication; and (4) system integration.

This work presents the preliminary steps and results of the prototyping process towards on-chip integration of an HCHO sensing system. This challenge was split into two individually addressed problems: (1) efficient airborne HCHO trapping in a microfluidic context and (2) low-concentration DDL molecular sensing in reduced interrogation volumes. Problem (1) was partially addressed and a gas–liquid microreactor concept was built around a disposable membrane-based PMMA flat contactor [16]. Problem (2) was addressed by proposing, fabricating, and testing a fluorescence detection system based on an ultrasensitive CMOS image sensor. Two three-layer fluidic cell configurations (*quartz*–SU-8–quartz and *silicon*–SU-8–quartz) were tested, with both possessing a 3.5 µL interrogation volume.

## 2. Materials and Methods

The fluorescence phenomenon is largely used for molecular optical sensing in microfluidic analytical applications due to its selectivity and capability to reach low detection limits, including single-molecule detection [25,26]. Fluorescence is the property of a molecule allowing it, once excited by light at a specific wavelength $\;\lambda_{abs}$, to emit light after some nanoseconds at a longer wavelength $\;\lambda_{em}$, a behavior known as the Stokes shift. A fluorescent molecule is characterized by the quantum yield  ϕ, defined as the ratio of the number of emitted photons to the number of absorbed photons. The number of photons emitted by fluorescence is usually up to three orders of magnitude less than the number of photons being absorbed to excite the molecule [27].

The amount of fluid involved in microfluidic systems is, by definition, reduced. Therefore, the emitted fluorescence signal in optofluidic sensors is very low. Traditional fluorescence detection systems use a complex optical path involving a system of lenses, a high-performance light source, and very sensitive photon detector to capture the low-intensity fluorescence signals [28]. Mainly due to these reasons, the systems are generally expensive, bulky, and barely portable in the best scenarios.

Miniaturization of fluorescence sensing optofluidic devices is a field currently experiencing intense research activity aiming to identify fabrication and design methodologies for an ultraportable, monolithic, and multiplexing sensing architecture. Advances in micro- and nanofabrication technologies have been used to develop new design strategies in order to maximize the detection while shrinking dimensions.

Therefore, when the design of an analytical microsystem based on fluorescence detection is attempted, the Stokes shift and the quantum yield of the molecule are fundamental prerequisites to be considered in order to properly identify the most suitable detection scheme, filtration elements, and light detector. The noise sources in optofluidic fluorescence-based sensors originate from both optical (shot and flicker components) and non-optical elements (dark noise of the detector) [29]. The optical background noise may originate from components with different spectra, e.g., other fluorophores, scattering light, and autofluorescence.

The DDL molecule has two absorption peaks: one in the ultraviolet (UV) range at the 255 nm wavelength and the other in the visible spectrum range at $\lambda_{abs}=412\;\mathrm{nm}$, while the fluorescence emission peak occurs at $\lambda_{em}=515\;\mathrm{nm}$, with a very low quantum yield ϕ(20 °C)=0.005 [30]. The shorter wavelength possesses a better fluorescence yield but a higher background signal. The absorption peak located at $\lambda_{abs}=412\;\mathrm{nm}$ was chosen for this prototype, since the UV light sources are more health damaging and less efficient from a cost perspective when compared to the violet light sources.

### 2.1. Complementary Metal-Oxide-Semiconductor (CMOS)-Based Fluorescence Detector: Sensor Design and Fabrication

**Figure 2:**
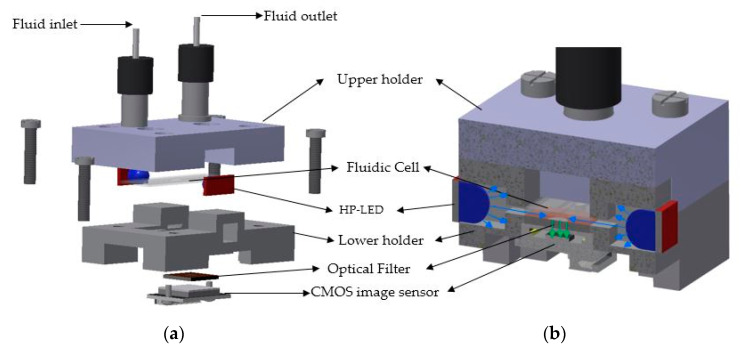
The concept of the complementary metal-oxide-semiconductor (CMOS)-based fluorescence detector (46 mm × 30 mm × 10 mm). (**a**) Exploded view of the detector. (**b**) Transversal cross-section of the assembled sensing device.

The concept of the optical detection system proposed in this work for the detection of the DDL molecules combined a couple of principles usually used in optofluidic sensing microsystems (see Figure 2). The design was based on an orthogonal detection scheme with a CMOS image sensor as the photon detector and high-power light emitting diodes (HP-LEDs) as excitation sources. A square band pass filter was selected and placed in between the fluidic cell and the CMOS image sensor. The components of both optical and fluidic circuits were hosted by two 3D-printed (Ultimaker 3 printer, Utrecht, The Netherlands) holders, named the upper and lower holders.

The particularity of this design relied on the orthogonal illumination scheme implemented in combination with the contact sensing principle. The contact sensing principle involves the photon detector, the filtration component (if it exists), and the fluidic cell being in direct contact. This is advantageous, since the optical path from the fluorescence source up to the photon detector is diminished, i.e., the optical loss is lower.

#### 2.1.1. Microfluidic Circuit

The fluidic system comprised the fluidic cell, the N-333 type connectors (IDEX Health and Science LLC, Rohnert Park, CA, USA), and the fluidic capillaries. The connectors were integrated into the upper holder. When the upper and lower holders were held together through the four screws, a leakage-free fluidic circuit was obtained, with an important role being played by the rubber sealing of the connectors.

**Figure 3:**
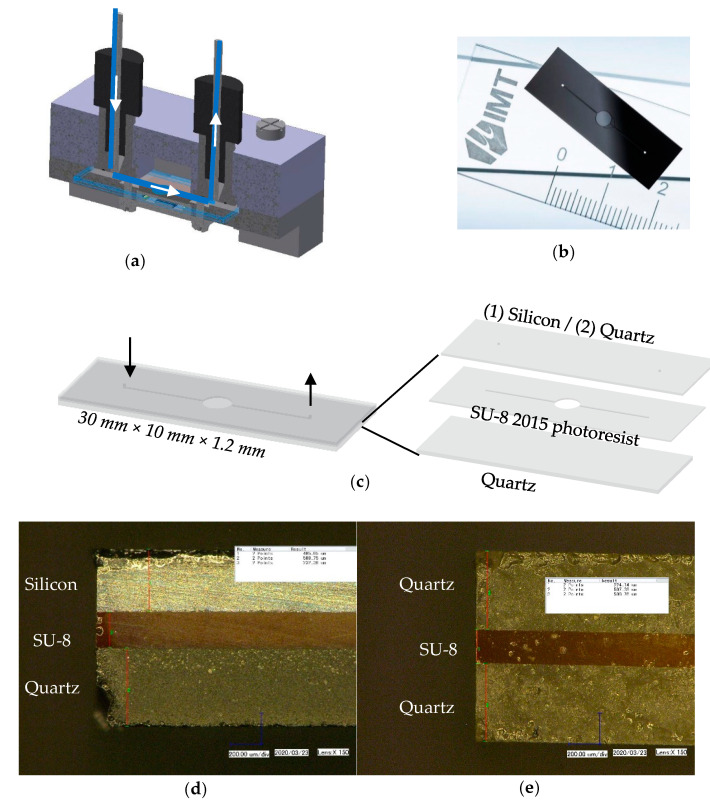
(**a**) Microfluidic circuit of the detection system. (**b**) Fabricated silicon fluidic cell. (**c**) Fluidic cell concept. (**d**) Microscope view of silicon (405 µm)–SU-8 (232 µm)–quartz (508 µm) fluidic cell cross-section. (**e**) Microscope view of quartz (507 µm)–SU-8 (224 µm)–quartz (508 µm) fluidic cell cross-section.

The fluidic cell, i.e., the element of the system hosting the microfluidic flow and the detection chamber from where the fluorescence signal is collected, was designed as a three-layer structure (Figure 3c) and fabricated using well-established photolithography techniques (Figure 3b,d,e).

Two fluidic cell configurations were fabricated, with the difference between the two consisting in the nature of the material of the upper layer. For the first configuration a quartz–SU-8 2015 negative photoresist–quartz structure (quartz fluidic cell) was employed, while for the second configuration a silicon–SU-8 2015 negative photoresist–quartz structure (silicon fluidic cell) was employed.

Quartz was selected due to its low autofluorescence emissions [31], this material being largely used for the fabrication of commercial flow-through fluidic cells. Silicon, a material largely used in the fabrication process of chip technology, possesses interesting properties for the particular aim of this project. More precisely, the absorption coefficient of silicon at the LED emission wavelength, $a\left(\lambda_{abs}=420\;\mathrm{nm}\right)=5\times 10^{4}{\;\mathrm{cm}}^{-1}$, is approximately five times higher than the absorption coefficient at the emission wavelength,$\;a\left(\lambda_{em}=515\;\mathrm{nm}\right)=9.25\times 10^{3}{\;\mathrm{cm}}^{-1}$ [32]. Therefore, the absorption of the excitation light in silicon, which contributes to the optical background noise, is expected to be larger than the absorption of the fluorescence emission, producing an enhancement of the detection limit of the system.

The fluidic cell (Figure 3b,d,e) was fabricated at the Laboratoire d’Analyse et d’Architecture des Systèmes (LAAS), Toulouse, France. A 4 inch 500 µm AF32 quartz wafer (Schott AG, Mainz, Germany) was initially cleaned with oxygen plasma at power P=800 W for t=5 min, followed by the deposition of a 200 µm SU-8 2015 negative photoresist layer (Kayaku Advanced Materials, Westborough, MA, USA) and baking using an EVG 120 machine (EVG, St. Florian am Inn, Austria). Afterwards, a mask was placed on top of the SU-8 layer, allowing UV light exposure using a Suss MA6 gen4 instrument (SÜSS MICROTEC SE, Garching, Germany) (λ=365 nm, t=42 s, power density $P_{d}=14\;\mathrm{mW}/{\mathrm{cm}}^{2}$) in order to create the desired channeling geometry, with a uniform 200 µm depth. Next, the structure was washed using an SU-8 developer to remove the exposed parts, then hard-baked (T=125 °C, t=1 min). The fluidic inlet and outlet were created through a piercing process on a second 500 µm quartz wafer. Initially, the quartz wafer was coated with Photec 2040 dry film (Hitachi Chemical, Tokyo, Japan) to protect the structure during the piercing process. The piercing was done by sandblasting, followed by rinsing and cleaning with acetone and deionized water. Then, the same oxygen plasma (P=800 W, t=5 min) cleaning procedure used for the first quartz wafer was repeated for the second one. An additional 10 µm SU-8 2015 negative photoresist layer was required on one side of the wafer to facilitate the bonding with the SU-8 2015 layer from the first wafer. Once this layer was coated, the two wafers were bonded using Nanonex NX Nanoimprint equipment (Nanonex, Monmouth Junction, NJ, USA) by applying uniform pressure on both sides. A dicing protection layer made of AZ 4562 photoresist layer (Microchemicals GmbH, Ulm, Germany) was coated and six fluidic cells were finally obtained with different interrogation volumes, as described by Mariuta et al. [33]. The geometry of the detection chamber (Figure 3c) was optimized using a computational fluid dynamics (CFD) model in Ansys Fluent to avoid fluid stagnation regions or dead volumes [33] and to match the sensing area ($4.8\times 4.8\;{\mathrm{mm}}^{2}$) of the photon detector.

#### 2.1.2. Optical Circuit

**Figure 4:**
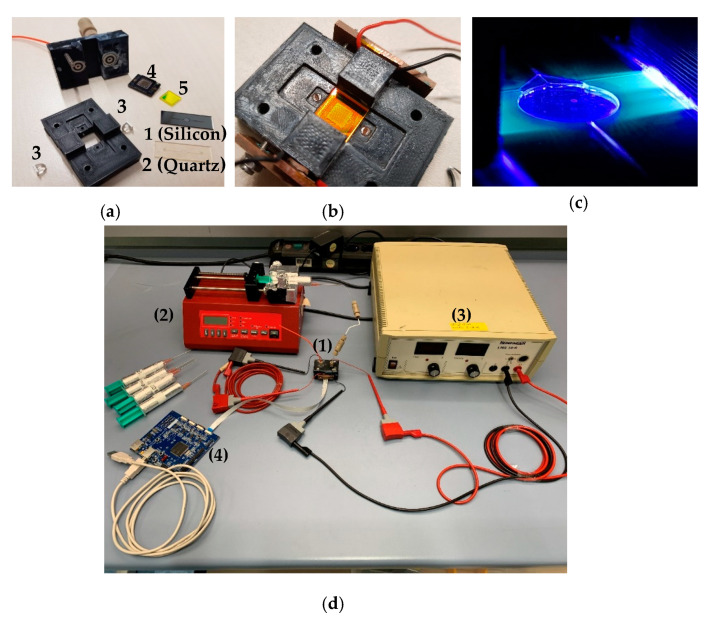
(**a**) Exploded view of the detection system before assemblage: (1) silicon fluidic cell; (2) quartz fluidic cell; (3) 420 nm light emitting diodes (LEDs); (4) CMOS image sensor; (5) band pass filter. (**b**) Assembled device;.(**c**) Light transmission through the fluidic cell towards the detection chamber. (**d**) (1) Experimental setup: assembled device, (2) syringe pump, (3) 24 V DC power supply, (4) microcontroller board.

The optical circuit of the sensing system relies on (see Figure 4): (1) the excitation light source; (2) the waveguide (SU-8 2015 negative photoresist layer); (3) the band pass filter; and (4) the CMOS image sensor.

LEDs are currently the most widespread excitation choice in microfluidic chemical sensors [34]. The current LEDs available on the market are high-power devices and are relatively cost-efficient. The main disadvantage of the LEDs when they are considered in optical sensing applications is related to the incoherence of the light beam, for which lasers perform better. LEDs are commercially available in the 210–3800 nm spectral range, with their emission spectrum being compatible with the molecules’ fluorescence excitation bands [34]. A SMB1N-420H-02 LED (Roithner Lasertechnik GmbH, Vienna, Austria) was selected for this prototype, with a maximal optical radiation power $P_{LED}=420\;\mathrm{mW}$ at $\lambda_{LED}=420\;\mathrm{nm}$ emission wavelength, viewing angle of 22°, with the maximum intensity concentrated at the center of its dispersion range. Two LEDs illuminated the detection chamber (see Figure 4a,b), considering its high diameter-over-depth ratio (23.5), to make sure that the excitation photons entirely penetrated the bulk of the interrogation volume.

Photomultiplier tubes (PMT), silicon photodiodes (SPD), CMOS image sensors, and more recently organic photodiodes (OPD) are photon detectors used in the miniaturization of fluorescence-based sensing devices. The recent evolution of the CMOS technology has introduced advantages, such as reduced dimensions, high sensitivity, coupling with filtration algorithms, very low prices per unit, low power consumption, and integrated signal processing, making it compatible for the miniaturization of fluorescence detection. On-chip CMOS integration, coupled with microfluidics for detection at the microscale, has increasingly been investigated in the recent literature [28,35,36,37], especially for the development of filterless prototypes.

A ULS24 (Anitoa Systems LLC, Menlo Park, CA, USA) CMOS image sensor model was selected as the photon detector for the current prototype, due to its ultra-low light sensitivity ( $3\times 10^{-4}$ lux), low-power supply (3.3 V/1.8 V, 30 mW), maximum sensitivity (60%) at $\lambda_{em}=515\;\mathrm{nm}$, and minimum sensitivity (40%) at $\lambda_{abs}=420\;\mathrm{nm}$. The CMOS image sensor was fabricated on a 0.18 µm CIS instrument with a die size of $4.8\times 4.8\;{\mathrm{mm}}^{2}$. The implemented “intelligent dark current management” algorithm made this image sensor capable of matching the sensitivity of the cooled charged-coupled device (CCD) and PMT technologies currently available on the market [38].

**Figure 5:**
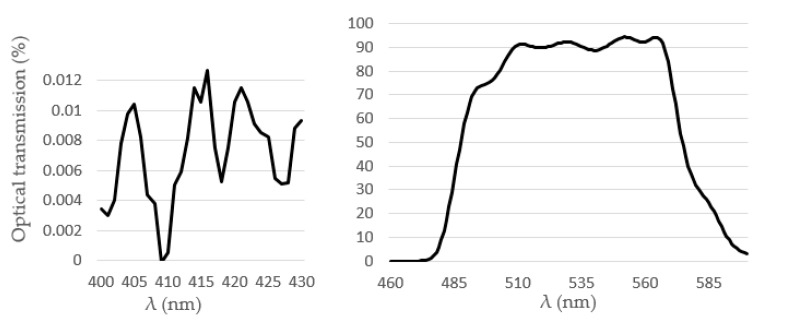
Optical transmittance of the band pass filter.

The transmission efficiency of the 540 ± 60 nm band pass filter, model BP525-R10, 1 mm thickness (Midwest Optical Systems, Palatine, IL, USA), was tested using a Perkinelmer Lambda 950 UV/VIS spectrometer (PerkinElmer, Waltham, MA, USA). It was found that the transmission efficiency was 0.01% at the excitation wavelength and 91.35% at the emission wavelength (see Figure 5). On top of the band pass filter, was placed a thin metallic layer (200 µm) with a central circular slit of 4.8 mm diameter in order to further minimize the amount of light and then reduce the optical noise induced by the excitation light reaching the CMOS photon detector.

### 2.2. CMOS-Based Fluorescence Detector: Experimental Set-Up

#### 2.2.1. Chemicals and Reagents

The Fluoral-P (0.01 M) was prepared by mixing 0.3 mL of acetic acid 100% (Merck, Molsheim, France), 0.2 mL of acetylacetone 99% (Merck), and 15.4 g of ammonium acetate 98% (Sigma-Aldrich, Lyon, France) in 200 mL 18.2 MΩ cm Milli-Q water at 25 °C (Millipore, Molsheim, France). The formaldehyde solutions ($c_{1}=0.02\;\mathrm{mg}/L$, $c_{2}=0.2\;\mathrm{mg}/L$, $c_{3}=2\;\mathrm{mg}/L$, $c_{4}=20\;\mathrm{mg}/L$) were prepared at the Institute of Chemistry and Processes for Energy, Environment and Health (ICPEES) Strasbourg by mixing a commercial formaldehyde solution (37% in water, Sigma-Aldrich) with Mili-Q water (18.2 MΩ·cm at 25 °C, Millipore). DDL solutions containing initial formaldehyde concentrations $(c_{1}=0.01\;\mathrm{mg}/L$,${\;c}_{2}=0.1\;\mathrm{mg}/L$,${\;c}_{3}=1.0\;\mathrm{mg}/L$,${\;c}_{4}=10.0\;\mathrm{mg}/L)$ were prepared just before the measurements by mixing Fluoral-P (0.02 M) and formaldehyde solutions (1:1 *v*/*v*), then placing it these an oven at T=65 °C for t=3 min to fully derivatize formaldehyde into DDL.

#### 2.2.2. Experimental Procedure

The two LEDs were connected in series, with the power being supplied from a 24 V DC source. The LEDs were turned on for short periods (t ≅ 1 s), while the data from the CMOS image sensor were captured using a PC interface during this activation time of the excitation source. Heat can cause a shift in the LED emission band, reducing the detection accuracy. Thus, the illumination of the detection chamber was done in short pulses (t ≅ 1 s) in order to avoid a temperature increase of the LEDs, which were provided with flat copper heat sinks. The interface provided a 12 × 12 matrix, representing the photon counts measured by each pixel, the temperature of the CMOS sensor at the measurement time, and a mean value $\bar{X}\;$ representing the average of all the $\;X_{i}$ 12 × 12 photon counts.

The ULS 24 CMOS image sensor was connected to the microcontroller board (Anitoa Systems LLC, Menlo Park, CA, USA), then to a personal computer (Figure 4d). The CMOS image sensor was settled to work in low-gain mode, 12 × 12-pixel configuration, and t=1 ms integration time.

The response of the system was iteratively tested for different DDL solutions at various formaldehyde concentrations ($c_{1}=0.01\;\mathrm{mg}/L$,$\;c_{2}=0.1\;\mathrm{mg}/L$,${\;c}_{3}=1.0\;\mathrm{mg}/L$,$\;c_{4}=10.0\;\mathrm{mg}/L$, and for the blank concentration $c_{0}=0.0\;\mathrm{mg}/L)$. During the measurement process, three different optical powers of the LED were employed: $\;P_{1}=0.25\;P_{LED}=105\;\mathrm{mW}$,$\;P_{2}=0.5\;P_{LED}=210\;\mathrm{mW}$,$\;P_{3}=0.75\;P_{LED}=315\;\mathrm{mW}$. The DDL solutions were continuously sampled during the measurements at a flow rate of 10 µL/min.

## 3. Results and Discussion

The experimental response of the system was evaluated by applying a regression analysis technique to the experimental data obtained from two independently prepared DDL samples (N=2, sample #1 and sample #2) for each of the tested concentrations, $\;c_{i,\;i=0,\ldots ,\;4}$. Each of the samples processed in the system was analyzed by taking seven continuous measurements (n=7) of the photon intensities, represented by $\;X_{i,\;i=1,\ldots ,\;7}$ in Equation (1). $\;X_{i}$ was in fact the mean value of all photon intensities given by the 12×12 pixel matrix obtained at the particular measurement time. Here, $\;{\bar{X}}_{\#1}$ represented the mean value of the $\;X_{i,\;i=1,\ldots ,\;7}$ measurements taken for the first sample, while $\;{\bar{X}}_{\#2}$ represented the mean value for the second sample investigated at a specific formaldehyde concentration of DDL solution (see Table 1 and Table 2).

The precision of the evaluation was quantified by calculating the absolute (σ) (Equation (1)) and relative standard deviations (RSD) (Equation (2)), and the performance was estimated using the signal-to-noise ratio (SNR) (Equation (3)). The SNR was used to describe the signal increase relative to the blank sample response ($c_{0}=0.0$ mg/L), but its relevance was limited due to the low number of independent samples tested. While $\sigma_{\#1}$ and $\sigma_{\#2}$ represented the standard deviations obtained for measurements taken for similar samples (${\bar{X}}_{\#1}\;$ and $\;{\bar{X}}_{\#2}$), σ represented the standard deviation of $\;{\bar{X}}_{N=2}=\left({\bar{X}}_{\#1}+{\bar{X}}_{\#2}\right)/2$ values.

(1) $$\sigma =\sqrt{\frac{\sum_{i=1}^{n}(X_{i}-\bar{X})}{N}},\;\mathrm{with}\;\bar{X}=\frac{1}{n}\sum_{i=1}^{n}X_{i}$$

(2) $$RSD=\frac{\sigma }{{\bar{X}}_{N=2}}$$

(3) $$SNR\left(c_{i}\right)=\frac{{\bar{X}}_{N=2}\left(c_{i}\right)-\bar{X}\left(c_{0}\right)}{\sigma \left(c_{i}\right)}$$

**Figure 6:**
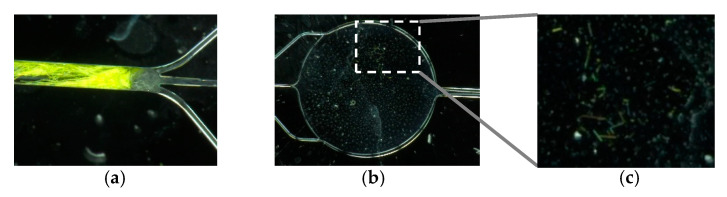
(**a**) Dry green DDL crystals cluster after streaming a DDL solution at a formaldehyde concentration of $\;c_{5}=100\;\mathrm{mg}/L$. (**b**) Detection chamber with (**c**) green DDL crystals on the inner walls after use of the DDL solution at a formaldehyde concentration of $\;c_{4}=10\;\mathrm{mg}/L$.

Memory effects (see Figure 6) were observed during the initial stages of the experimental campaign. Therefore, the fluidic cells were rinsed with acetone and dried in the oven after each sampling.

**Figure 7:**
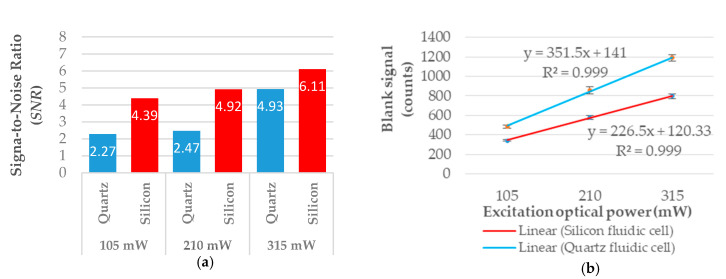
(**a**) Signal-to-noise ratios (SNRs) obtained for quartz and silicon fluidic cells at a formaldehyde concentration of $\;c_{1}=0.01$ mg/L in DDL solution and with different optical powers. (**b**) The linear response obtained for three excitation optical powers at $c_{0}=0.00$ mg/L (blank sample). The error bars indicate the standard deviations for two independently prepared samples (N=2).

**Figure 8:**
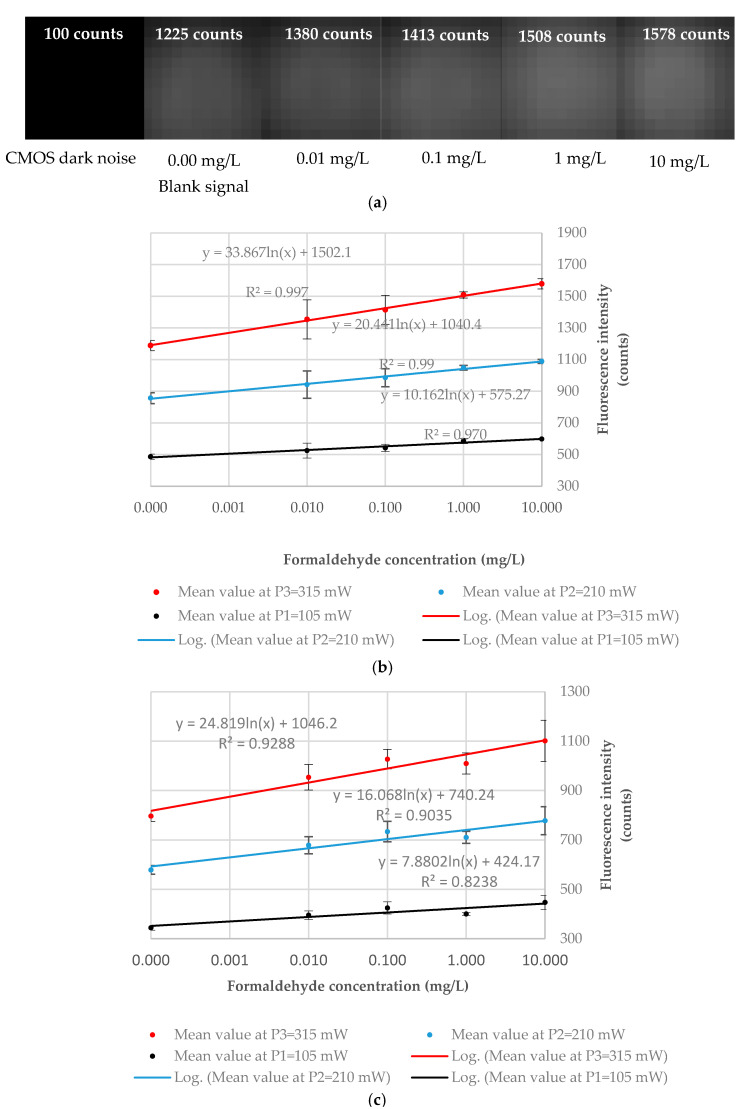
Experimental evaluation of the CMOS-based fluorescence detector realized using the mean values ($\bar{X}$) of seven measurements (n=7 ) made on two independently prepared samples (N=2 ) at each of the $c_{i},\;i=0,\ldots ,\;4\;$ concentrations tested. (**a**) ULS 24 CMOS image sensor 12 × 12-pixel output for the dark current, blank signal, and the four formaldehyde concentrations tested for a quartz cell at *P*=315 mW. (**b**) Logarithmic fitting of the experimental points for a quartz fluidic cell. (**c**) Logarithmic fitting of the experimental points for a silicon fluidic cell. The error bars indicate one standard deviation (N=2 ).

The response of the CMOS-based fluorescence detector for a blank sample ($c_{0}=0.00$ mg/L) was plotted in Figure 7b. By increasing the excitation optical power, a perfect linear response (R2=0.999) was obtained for both quartz (y=351.5 x+141) and silicon (y=226.5 x+120.3) cells. However, a higher steepness of the linear equation was observed for the quartz fluidic cell compared to the silicon fluidic cell. Moreover, lower values of the photon counts were measured for the silicon cell. The lower intensity and steepness measured for the silicon cell may have been caused by two main factors: (1) lower excitation light intensity reaching the detection chamber, and consequently the photon detector, due to the 400 µm thickness of the opaque silicon upper layer; (2) the light absorbance of silicon at $\;\lambda_{abs}=420\;\mathrm{nm}$ is approximately five times higher than the absorbance of silicon at $\;\lambda_{em}=515\;\mathrm{nm}$. This latter factor might also explain the differences between the SNRs obtained for the two fluidic cell configurations (see Table 1 and Table 2, Figure 7a). Analyzing the results obtained for both fluidic cells (Figure 8b,c), it was observed that the fluorescence intensity measured for the silicon cell was generally lower compared to that of the quartz cell.

From the presented experimental dataset, relatively high values of the photon counts were observed for the blank sample (see Figure 7b). This was mainly due to the fact that the transmission efficiency of the band pass filter at the excitation wavelength was not exactly zero (see Figure 4). This fact caused 0.008% of the excitation optical signal to reach the very sensitive ULS24 CMOS image sensor.

The CMOS image sensor response as a 12 × 12 light intensity matrix representation is illustrated in Figure 8a. The image corresponding to the CMOS dark noise depicted its response in a complete dark environment (no excitation light). The second picture from the left to the right illustrated the CMOS image sensor response for a 0.0 mg/L concentration solution (blank sample), with a value of 1225 counts being registered. As expected, higher values were obtained once the DDL concentration streamed into the system was iteratively increased. Due to the fact that the differences between the pixel outputs were relatively low compared to the dynamic range of the image sensor, the gray color gradient found was also low. However, there was a visible slight enhancement in the gray color intensity from the left to the right coming from the circular detection volume.

The experimental response of the CMOS-based fluorescence detector presented in Figure 8b,c shows a logarithmic correlation between the DDL fluorescence intensity and formaldehyde concentrations in DDL solutions. Analyzing the plots, it was observed that the correlation coefficients ($R^{2}$) increased proportionally with the excitation optical power for both cells. The logarithmic behavior suggested that the concentration range tested was above the limit of linearity of the system [39]. A logarithmic behavior of the fluorescence signal was observed in the 0.01–10 mg/L formaldehyde concentration range for both cells, indicating that the upper limit of the linear range is most probably below the lowest concentration tested, i.e., 0.01 mg/L. The RSD calculated for measurements performed on a particular sample gave values ranging from 0.06% up to 0.71% for the quartz cell (see Table 1) and values ranging 0.09% to 1.59% for the silicon cell (see Table 2). When RSDs were calculated for the duplicate samples, values ranging from 1.29% to 9.11% for the quartz cell and from 3.82% to 7.54% for the silicon cell were found. The source of errors might be related to the inherent differences that arose during the preparation of the samples, the uncontrolled temperature of the environment, and the low number of replicates. Moreover, bubbles were sometimes observed at the end of the measurement process inside the fluidic cell detection chamber. The observed fluorescence intensity mean values were higher when bubbles were present inside the chamber compared to the situation when bubbles were absent. This is why the measurements made in the presence of air bubbles occupying parts of the detection chamber were not considered when the response of the sensor was determined. The sources of error mentioned above might be the reason why the mean values obtained for the silicon fluidic cell at ${\;c}_{3}=1.0$ mg/L were observed as being lower than expected for all the three optical excitation powers tested (see Figure 8c). Analyzing the plot given by the other three concentrations, it could be observed that the values obtained at this concentration were below the trend line; moreover, the values obtained at ${\;c}_{3}=1.0$ mg/L were lower than the values obtained at ${\;c}_{3}=0.1$ mg/L.

Although the results obtained were approximate, a tendency toward higher SNRs in the case of the silicon fluidic cell could be noted. The SNR obtained for the lower formaldehyde concentration tested, ${\;c}_{1}=0.01$ mg/L, is plotted in Figure 7a. At the maximum optical power tested, the SNR value of the quartz cell was found to be $\;SNR_{quartz}=4.9$, while $\;SNR_{silicon}=6.1$.

## 4. Conclusions and Perspectives

In conclusion, it should be noted that the main achievements registered in this work were related to the on-chip integration of the detection part of the sensor. A CMOS-based fluorescence detector was developed and proved to be capable of detecting 10 µg/L of formaldehyde derivatized into DDL (ϕ(20 °C)=0.005) in a 3.5 µL interrogation volume with  SNR=4.9 and RSD=9.1% (N=2) for a quartz fluidic cell; and with  SNR=6.1 and RSD=5.3% (N=2) for a silicon fluidic cell. Moreover, the enhancement of the signal intensity in the silicon fluidic cell due to its absorption coefficient at the LED emission wavelength,$\;a\left(\lambda_{abs}=420\;\mathrm{nm}\right)=5\times 10^{4}{\;\mathrm{cm}}^{-1}$, which is approximately five times higher than the absorption coefficient at the emission wavelength,$\;a\left(\lambda_{em}=515\;\mathrm{nm}\right)=9.25\times 10^{3\;}{\mathrm{cm}}^{-1}$, was confirmed.

Although the repeatability of the results has to be tested for more independently prepared samples in order to fully validate the system, the potential of the concept has been demonstrated through the obtained results. The logarithmic behavior of the fluorescence signal was observed in the 0.01–10 mg/L formaldehyde concentration range for both cells, indicating that the upper limit of the linear range is most probably below the lowest concentration tested, i.e., 0.01 mg/L. The linear range of the system could be obtained after lowering the optical background noise level, which was observed as being relatively high.

Otherwise, the feasibility of the detection configuration has been proven, with the system showing high miniaturization and detection improvement potential. The 1 mm thickness band pass filter could be replaced by a filtration layer coated directly on top of the CMOS image sensor, as described by Guduru et al. [40] and Sunaga et al. [41]. This would further diminish the optical path length and lead towards a fully-integrated monolithic device. Moreover, a filterless configuration based on time-resolved detection methodology could be employed, as described by Mariuta et al. [16]. This methodology practically avoids the optical background noise, but its implementation is strictly related to the working frequency of the processor involved, since the DDL molecule fluorescence decay time is only limited to 2 ns.

The study showed the functionality of the concept based on an orthogonal detection scheme, employing a LED-induced fluorescence technique coupled to a CMOS image sensor as an ultra-low light detection system. Due to the memory effects that were consistently observed, the fluidic channels were rinsed with acetone after each sampling.

In order to further reduce the fabrication costs, PMMA polymer, providing very good optical properties for this particular application, might be considered as the main material for the fabrication and testing of a new fluidic cell. Its implementation as a waveguide for biomedical applications and its potential for wearable and in vivo applications, which are currently being studied [42], makes it a promising material for such an application. Here, attention has to be paid to the surface roughness that results from the fabrication process, possible swelling from liquids, and its own fluorescence. Another possible direction could be represented by a full silicon structure, considering the CMOS image sensor validation for this application and the SNR enhancement observed for silicon.

In addition, the potential of this device could go beyond this specific application, contributing to the rapidly developing field of on-chip fluorescence sensing for various applications of rapid and low-cost monitoring in chemistry, biology, or environmental fields.

Regarding the challenge related to airborne HCHO trapping in a microfluidic context, a gas–liquid microreactor concept was proposed [16]. A disposable gas–liquid microcontactor, designed as a two-layer PMMA structure with an embedded polymeric membrane, is supposed to be integrated in between two holders equipped with an integrated heating and fluidic streaming system. Each PMMA layer of the contactor hosts a meandering network of rectangular cross-section microchannels, with one layer being assigned to the reagent streaming (liquid carrying layer) and one to the gas streaming (gas carrying layer).

After successful fabrication of the subsystems of the gas–liquid microreactor, further results are expected to experimentally prove the concept. Further analytical studies have to be performed in order to check the viability of the on-chip membrane integration technique and to study the mass transfer efficiency at different flow rates of gas and reagent streams. A mathematical model for the estimation of formaldehyde trapping in the reagent stream through a hydrophobic membrane was also developed and presented by Mariuta et al. [16]. The interest here is to enable enhanced and efficient formaldehyde trapping using cost-efficient on-chip membrane-based polymer chips. Next, a system integration study has to be performed in order to identify solutions for low-noise, cost-effective fluid pumping.

## Figures and Tables

**Table 1:** Error distribution as the standard deviation (σ), relative standard deviation (RSD), and the signal-to-noise ratio (SNR) for the quartz fluidic cell at the excitation optical power,  P=315 mW, where #1 and #2 represented the two independently prepared samples for each of the tested formaldehyde concentrations $\;c_{i}\;\mathrm{for}\;i=0,\ldots ,\;4$.

Formaldehyde Concentration (mg/L)	0.00	0.01	0.10	1.0	10.0
${\bar{X}}_{\#1}$ (counts)	1122	1477	1505	1528	1611
$\sigma_{\#1}$	3	2	3	4	5
$\;RSD_{\#1}\;\left(\%\right)$	0.21	0.16	0.21	0.29	0.29
${\bar{X}}_{\#2}$ (counts)	1155	1230	1323	1489	1546
$\sigma_{\#2}$	3	9	2	1	3
$\;RSD_{\#2}\;\left(\%\right)$	0.24	0.71	0.16	0.06	0.20
${\bar{X}}_{N=2}$ (counts)	1189	1354	1414	1509	1578
$\;{\bar{X}}_{N=2}$	34	123	91	20	33
*RSD* (%)	2.82	9.11	6.43	1.29	2.06
*SNR*	-	4.9	6.7	9.6	11.6

**Table 2:** Error distribution as the standard deviation (σ), relative standard deviation (RSD), and signal-to-noise ratio (SNR) for the silicon fluidic cell at the excitation optical power  P=315 mW, where #1 and #2 represented the two independently prepared samples for each of the tested concentrations $\;c_{i}\;\mathrm{for}\;i=0,\ldots ,\;4$.

Formaldehyde Concentration (mg/L)	0.00	0.01	0.1	1.0	10
${\bar{X}}_{1}$ (counts)	771	902	988	966	1,018
$\sigma_{\#1}$	3	1	2	15	2
$\;RSD_{\#1}\;\left(\%\right)$	0.36	0.15	0.24	1.59	0.22
${\bar{X}}_{2}$ (counts)	822	1005	1066	1053	1184
$\sigma_{\#2}$	4	8	2	2	1
$RSD_{\#2}\;\left(\%\right)\;$	0.44	0.75	0.21	0.16	0.09
${\bar{X}}_{N=2}$ (counts)	797	954	1027	1010	1101
$\;{\bar{X}}_{N=2}$	26	51	39	43	83
*RSD (%)*	3.21	5.39	3.82	4.27	7.54
*SNR*	-	6.1	9.0	8.3	11.9
